# Decreased serum testosterone level was not significantly correlated with lipid indices in elderly men

**DOI:** 10.22088/cjim.12.2.135

**Published:** 2021-03

**Authors:** Neda Meftah, Ali Bijani, Seyed Reza Hosseini, Amir Morteza Soleimani

**Affiliations:** 1 Clinical Research Development Unit of Rouhani Hospital, Babol University of Medical Sciences, Babol, Iran; 2 Social Determinants of Health Research Center, Health Research Institute, Babol University of Medical Sciences, Babol, Iran; 3 Student Committee Research, Babol University of Medical Sciences, Babol, Iran

**Keywords:** Testosterone, Men, Lipid indices, Hypogonadism

## Abstract

**Background:**

Aging in men causes a gradual decline in endogenous testosterone levels, which may have detrimental effects on their health status. Testosterone deficiency is thought to promote atherosclerosis by modulating lipid metabolism. Therefore, this study was conducted to evaluate the serum testosterone level and its correlation with lipid profile in men aged ≥60 years old.

**Methods:**

All elderly men aged ≥60, residing in Amirkola and participating in a phase of the comprehensive project on "investigating the health status of the elderly in Amirkola" were entered into this descriptive cross-sectional study. After fasting over 12 hours, the venous blood samples were taken. Serum concentration of testosterone was determined using ELISA method. Moreover, HDL-LDL, total cholesterol, triglyceride and fasting blood glucose were measured.

**Results:**

The prevalence of hypogonadism was 91.28% among the 792 participants of this study with a cut-off point of 9.72 nmol/L (95% confidence interval, 93.25-89.31) and the prevalence of severe hypogonadism with a cut-off point of 5.2 nmol/L was reported 71.59% (95% confidence interval, 74.73-68.44%). Based on the results, there was no significant statistical correlation between the serum level of testosterone and triglyceride (r=0.03, P=0.34). Furthermore, there was a negative correlation between testosterone and HDL, which was not statistically significant(r=-0.05, P=0.13). No significant statistical correlation was found between testosterone and LDL (P=0.98). There was a negative correlation between testosterone and cholesterol, which was not statistically significant (r=-0.02, P=0.49).

**Conclusion:**

According to the study results, 91% of men aged ≥60 years old had hypogonadism, no correlation was found between testosterone and lipid indices.

Testosterone is the main sex hormone in man. A young man produces 3–10 mg of testosterone daily that results in serum levels of 300–1000 ng per 100 ml. Testosterone level is generally lower in the late afternoon and is suppressed by glucose administration or food intake (1). Although considering specific testosterone threshold is likely to be unreliable, generally, the symptoms and signs of androgen insufficiency more likely occur with total testosterone level lower than normal for young healthy men (less than 280-300 ng/dl or 9.7-10.4 nmol/L (2). Epidemiological studies have displayed that total and free testosterone levels decline with aging. Clinical outcomes of male hypogonadism are well known. 

These include fatigue, erectile dysfunction, loss of muscle mass and strength, increased fat mass, reduced sexual desire and activity, low energy and depression (3-6). In addition, a decrease in testosterone level is associated with an increase in the serum estrogen level due to increased aromatase activity. Consequently, the increased estrogen level enhances serum cholesterol and atherogenic lipoprotein particles (7). Recent investigations have demonstrated that low testosterone levels are associated with atherosclerosis in all major vessels (8). Moreover, animal experiments have indicated that testosterone inhibits atherosclerosis plaque formation in rabbits and rodents fed a high-fat diet (9). In line with these evidence, another study showed that men with ischemic heart disease had lower levels of testosterone than controls and that serum testosterone levels were conversely correlated to the degree of coronary atherosclerosis (9). However, lipid metabolism disorder plays a major role in atherosclerotic plaque formation, whether testosterone deficiency is a risk factor for dyslipidemia or whether the replacement of testosterone is useful for men ≥60 years old or not is still one of the major challenges that have not been answered conclusively. Therefore, this study was designed to evaluate the serum testosterone level and its correlation with lipid profile in elderly men.

## Methods

**Study population and sampling: **All elderly people ≥60 years old residing in Amirkola and participating in a phase of the comprehensive project on "investigating the health status of the elderly in Amirkola" were entered into this descriptive cross-sectional study. The plan implementation and its importance were fully explained to the patients and written informed consent was obtained from each of them. Patients with acute illness at the time of sampling, pituitary or hypothalamus diseases, renal failure, cancer and history of consumption steroids**, **synthetic androgen, opioids**, **anticonvulsant drugs were excluded from the current study (8). But patients with current statin consumption were not excluded. The correlation between testosterone and lipid was determined based on the use or no use of statins. The venous blood sample was taken from patients at 8-10 a.m. after fasting over 12 hours. The serum was collected and stored at -80 °C until analysis. 

**Measurement of serum testosterone by ELISA: **Serum concentration of testosterone was determined using ELISA method. In this study, according to other studies, the reference range for total testosterone was from 19.72 to 27.76 nmol/l (9). Accordingly, patients were assigned into two groups of normal and low testosterone, In this study we defined the hypogonadodism as: a patient who has a testosterone level less than 280 ng/dl or 9.7 nmol/l and patients with serum testosterone level <5.2 nmol/l were considered as severe hypogonadism.

**Measurement of lipid indices by biochemical investigation: **Fasting plasma glucose, serum total cholesterol (T-Cho), triglycerides (TG), HDL-Cho and LDL-Cho were estimated by enzyme-based colorimetric methods using commercial kits. For dyslipidemia, the parameters were defined as hypercholesterolemia for total cholesterol >240 mg/dl, as hypertriglyceridemia for fasting triglyceride >220 mg/dl, as high LDL for LDL >160 mg/dl and as low HDL for HDL <40 mg/dl.

**Statistical analysis: **Data were analyzed using SPSS 22. Chi-square test, t test, Pearson correlation and regression model were used and a p-value <0.05 was considered significant. In addition, comparison of lipid disorders was performed between those with normal testosterone level and those with testosterone deficiency. Homogenization was done in groups through regression model. The effect of factors involved in lipid profile such as diabetes was evaluated on two groups with low and normal testosterone levels and on the decrease or increase of dyslipidemia. Multivariate analysis was performed to eliminate confounding factors such as BMI, fasting blood sugar and blood pressure.

## Results

In the current study, the mean testosterone level of 792 men aged ≥60 participated in the Amirkola Cohort project was 4.77±4.10 with a range of 10.04-34.90 nmol. The prevalence of hypogonadism was 91.28% among the 792 participants of this study with a cut-off point of 9.72 nmol/L (95% confidence interval, 93.25-89.31) and the prevalence of severe hypogonadism with a cut-off point of 5.2 nmol/L was reported 71.59% (95% confidence interval, 74.73-68.44%).

Based on the obtained results, there was no significant statistical correlation between serum testosterone level and triglyceride (r=0.03, P=0.34). In addition, there was a negative correlation between testosterone and HDL, which was not statistically significant (r=-0.05, r=-0.13). No significant statistical correlation was also found between testosterone and LDL (P=0.98). There was a negative correlation between testosterone and cholesterol, which was not statistically significant (r=-0.02, P=0.49). 

In general, statins were used by 451 (56.5%) of 792 old patients. The correlation between testosterone and lipid profile in the elderly was determined based on the use or no use of statins. There was a negative correlation between testosterone and triglyceride in the elderly who took statin, which was not statistically significant (r=-0.02; r=-0.55). Furthermore, there was a negative correlation between testosterone and HDL (r=-0.07, P=0.12), between testosterone and LDL (r=-0.07, P=0.12) as well as between testosterone and cholesterol (r=-0.07, P=0.13), which was not statistically significant. There was a significant positive correlation between testosterone and triglyceride in the elderly who did not take statins (r=0.12, p=0.02). In other words, increasing the level of one factor escalates the other factor. A negative correlation was found between testosterone and HDL, which was not statistically significant (r =-0.03, P=0.57). Moreover, there was no significant statistical correlation between testosterone and LDL (r=0.10, P=0.051) as well as testosterone and cholesterol (r=0.04, p=0.36) 

In the study of serum testosterone level with different lipid indices, it was found that the mean testosterone level had no statistically significant difference in various ranges of lipid indices. The mean serum testosterone level was categorized with cut-off points of ≤9.72 nmol/l and >9.72 nmol/l and was compared with the lipid profile (table 2). In terms of correlation between serum testosterone level of 9.72 and ranges of lipid indices, no significant correlation was found between testosterone of 9.72 and lipid factors. The mean serum testosterone level was categorized with cut-off points of ≤5.2 nmol/l and >5.2 nmol/l and was compared with the lipid profile. In terms of correlation between serum testosterone level with a cut-off point of 5.2 nmol/l and ranges of lipid indices, no significant correlation was found between lipid factors and severe hypogonadism.

## Discussion

Testosterone deficiency (TD) in aging men is defined existence of both clinical symptoms and low serum testosterone levels as we approach in younger or middle-aged men, because lack of high-quality, randomized trials in older men, diagnosis of TD in older men is more difficult due to the existence of symptoms associated with normal aging that overlap with those associated with TD. However, evaluating patients for biochemical TD alone can lead to significant over-diagnosis due to age-related decline in testosterone levels. In fact, many older men with ‘low testosterone’ are asymptomatic. This problem has led many large medical societies and organizations to define the phenomenon of ‘late-onset’ hypogonadism (LOH) or andropause as a ‘clinical and biochemical syndrome associated with advancing age ,LOH characterized by symptoms and a deficiency in serum testosterone levels below those seen in young healthy males’ . For these reasons, the apparent low testosterone may be a normal physiologic response with aging (10, 12). In the present study, 91.28% of men >60 years had hypogonadism, among them 71.6% had severe hypogonadism.

 Recent evidence has indicated that low serum testosterone levels is a risk factor for diabetes, metabolic syndrome and inflammation. In addition, there are several conflicting investigations regarding the association between low testosterone levels and atherogenic lipid profile (11). However, the detailed effect of testosterone on dyslipidemia or whether testosterone replacement is useful for elderly men is still unclear. Therefore, this study was conducted to evaluate the serum testosterone level and its correlation with lipid profile in men aged ≥60. 

The most important finding of this study was the lack of correlation between serum testosterone level and lipid indices. In a study by Granato et al., they investigated the role of testosterone, lipid metabolism and epicardial fat thickness in patients with Klinefelter syndrome, there was no correlation between testosterone and lipid in the control and patient (with syndrome) groups, which is consistent with the finding of the present study. In addition, there was an inverse correlation between testosterone and epicardial fat thickness. In other words, the epicardial fat thickness was reduced with increased serum testosterone level (13). In contrast to our findings, shen et al. demonstrated that there was a relationship and correlation between testosterone and lipid profile. Furthermore, they suggested that the low serum level of testosterone could be a risk factor for increasing cholesterol and LDL levels (14). Similarly, Abdelazez et al. indicated that the increased level of testosterone would raise HDL and decrease cholesterol and LDL levels (15). A systematic and meta-analytical study conducted by Zhang et al. showed that total cholesterol and TG levels declined through testosterone treatment in men with hypogonadism and type 2 diabetes. They also demonstrated that hormone therapy with testosterone could increase HDL level (16). In another study by Zhang et al., there was a significant inverse correlation between serum total testosterone level with total cholesterol, TG and LDL, while serum testosterone level had a significant direct correlation with HDL (9). 

The possible reasons for these conflicting results might be due to the effect of underlying diseases in older men. Because some of these diseases can affect the amount of testosterone. In addition, the total testosterone was evaluated in the present study, whereas the free testosterone as well as total testosterone was assessed in the above-mentioned studies. To further expand that, Shen et al. suggested that free testosterone was lower than total testosterone in older men. They declared that the free testosterone might be a better marker to diagnose the age-related androgen deficiency in men (17). Vodo et al. also found that the testosterone level had an inverse relationship with total cholesterol, LDL and TG (18-20). Moreover, Bica et al. concluded that the testosterone level significantly had a direct correlation with HDL level and an inverse correlation with TG (20). 

Although various studies performed on healthy individuals have shown that some metabolic diseases cause changes in sex hormones and, consequently changes blood lipids and risk factors for cardiovascular diseases (22), none of these studies are conclusive and their results are contradictory with each other. Wickramatilake et al. represented that in men with coronary artery disease, there was a significant direct correlation between testosterone and HDL, but a there was a significant inverse correlation between testosterone and LDL (23). Although the underlying disease of males has not been investigated in the present study, due to their high age, it can be expected that there is a history of disease in males >60 years of age. In another study, Anderson et al. illustrated that among healthy individuals, the increased testosterone level led to a decrease in serum HDL level, but had no effect on total cholesterol, triglyceride and LDL levels (24). 

One of the strengths of this study was the investigation of correlation between testosterone and lipid indices in men who used statins and men who did not use statins. Testosterone correlation with individual lipid indices was assessed in two groups, but there was no significant correlation between two groups. Only in men who did not take statins, there was a positive correlation between testosterone and triglyceride, meaning that increasing the level of each of these two factors enhances the level of the other factor. In the present study, the variables were evaluated with two cut-off points of 9.72 and 2.5 nmol/l testosterone. The lipid profile had no significant difference with serum testosterone level at two cut-off points. Based on the results of the current study, the prevalence of hypogonadism in males aged ≥60 was 91%, and no correlation was found between testosterone and lipid indices. In conclusion, it should be noted that the relationship between testosterone and various lipid indices is different in various studies (direct relationship in some and inverse in other studies). Many factors such as ethnicity, lifestyle and different comorbidities may justify this discrepancy.

So these inconsistencies indicate that there is still a need for further studies in this field to thoroughly examine and clarify a certain conclusion before androgen replacement in men with low levels of testosterone and suggesting that the age-related total testosterone decline may be at least partly prevented through the management of potentially modifiable risk factors and health related behavior.

## Funding:

None

## Conflict of interest:

 The authors declare no conflict of interest.
